# Retinal Ganglion Cell Loss Is Accompanied by Antibody Depositions and Increased Levels of Microglia after Immunization with Retinal Antigens

**DOI:** 10.1371/journal.pone.0040616

**Published:** 2012-07-26

**Authors:** Stephanie C. Joachim, Oliver W. Gramlich, Panagiotis Laspas, Heiko Schmid, Sabine Beck, Harald D. von Pein, H. Burkhard Dick, Norbert Pfeiffer, Franz H. Grus

**Affiliations:** 1 Experimental Eye Research Institute, Ruhr University Eye Hospital, Bochum, Germany; 2 Experimental Ophthalmology, Department of Ophthalmology, University Medical Center, Johannes Gutenberg University, Mainz, Germany; 3 Department of Neuropathology, University Medical Center, Johannes Gutenberg University, Mainz, Germany; University of Rochester, United States of America

## Abstract

**Background:**

Antibodies against retinal and optic nerve antigens are detectable in glaucoma patients. Recent studies using a model of experimental autoimmune glaucoma demonstrated that immunization with certain ocular antigens causes an immun-mediated retinal ganglion cell loss in rats.

**Methodology/Principal Findings:**

Rats immunized with a retinal ganglion cell layer homogenate (RGA) had a reduced retinal ganglion cell density on retinal flatmounts (p = 0.007) and a lower number of Brn3^+^retinal ganglion cells (p = 0.0001) after six weeks. The autoreactive antibody development against retina and optic nerve was examined throughout the study. The levels of autoreactive antibodies continuously increased up to 6 weeks (retina: p = 0.004; optic nerve: p = 0.000003). Additionally, antibody deposits were detected in the retina (p = 0.02). After 6 weeks a reactive gliosis (GFAP density: RGA: 174.7±41.9; CO: 137.6±36.8, p = 0.0006; %GFAP^+^ area: RGA: 8.5±3.4; CO: 5.9±3.6, p = 0.006) as well as elevated level of Iba1^+^ microglia cells (p = 0.003) was observed in retinas of RGA animals.

**Conclusions/Significance:**

Our findings suggest that these antibodies play a substantial role in mechanisms leading to retinal ganglion cell death. This seems to lead to glia cell activation as well as the invasion of microglia, which might be associated with debris clearance.

## Introduction

The pathogenesis of glaucoma is likely influenced by several factors. High intraocular pressure is known to be not solely responsible for the disease. Other possible pathogenic components, such as apoptotic processes [1], [2], elevated nitric oxide levels [3] or involvement of the immune system [4] have received increased interest. Our group and others could identify antibody pattern alterations against retina and optic nerve in glaucoma patients [5], [6], [7]. Antibodies against ocular antigens, such as heat shock proteins [8], [9], γ-enolase [10], or α-fodrin [11], are possible factors in disease development. These findings support the hypothesis of an autoimmune component in glaucoma. So far, the question whether changes in antibody reactivities are a trigger of retinal ganglion cell (RGC) loss or simply an epiphenomenon of the disease remains unanswered.

In general, the mammalian retina contains different types of neuron-supporting macroglia cells, astrocytes and Müller cells [12], as well as microglia cells. Microglia are located in the nerve fiber layer, ganglion cell layer, and inner plexiform layer [13], [14]. Glaucomatous eyes demonstrate an increase of GFAP immunoreactivity [15], which could be a cellular attempt to foster tissue repair and hinder neuronal injury. Tezel et al. detected an enhanced immunostaining of GFAP in Müller cells and astrocytes of human glaucoma eyes as well as an increased number of microglia [16]. In the ocular hypertension model (OHT) a continuous increase in GFAP immunoreactivity can be observed [17], likely as a response to stress and RGC damage [18]. Reactive glia is the common hallmark of CNS injury and also microglia known to react to traumatic cell death [19]. Activation of microglia was noted in retina and optic nerve of OHT animals [20]. The greater the degree of optic nerve injury in this model, the greater the number of microglia. Activation of microglia has also been described in experimental autoimmune encephalitis (EAE), an animal model of multiple sclerosis [21], [22], where they seem to play a crucial role in disease development.

It is still not known if antibodies against retinal antigens detected in glaucoma [6], [7] are cause or consequence of this disease. First studies using a model of autoimmune glaucoma provided evidence that immunization with specific heat shock proteins leads to RGC loss [23], [24]. In this study, we aimed to find out if the RGC degeneration in this model is accompanied by the development of autoreactive antibodies against ocular structures or alterations in glia cell levels. We analyzed the effects of immunization with an antigen mixture of ganglion cell-layer proteins on retinal ganglion and glial cells. Further, we examined the occurrence of ocular autoreactive antibodies to gain more knowledge about the significance of antibodies in RGC death.

## Results

### Observations

**Figure 1:**
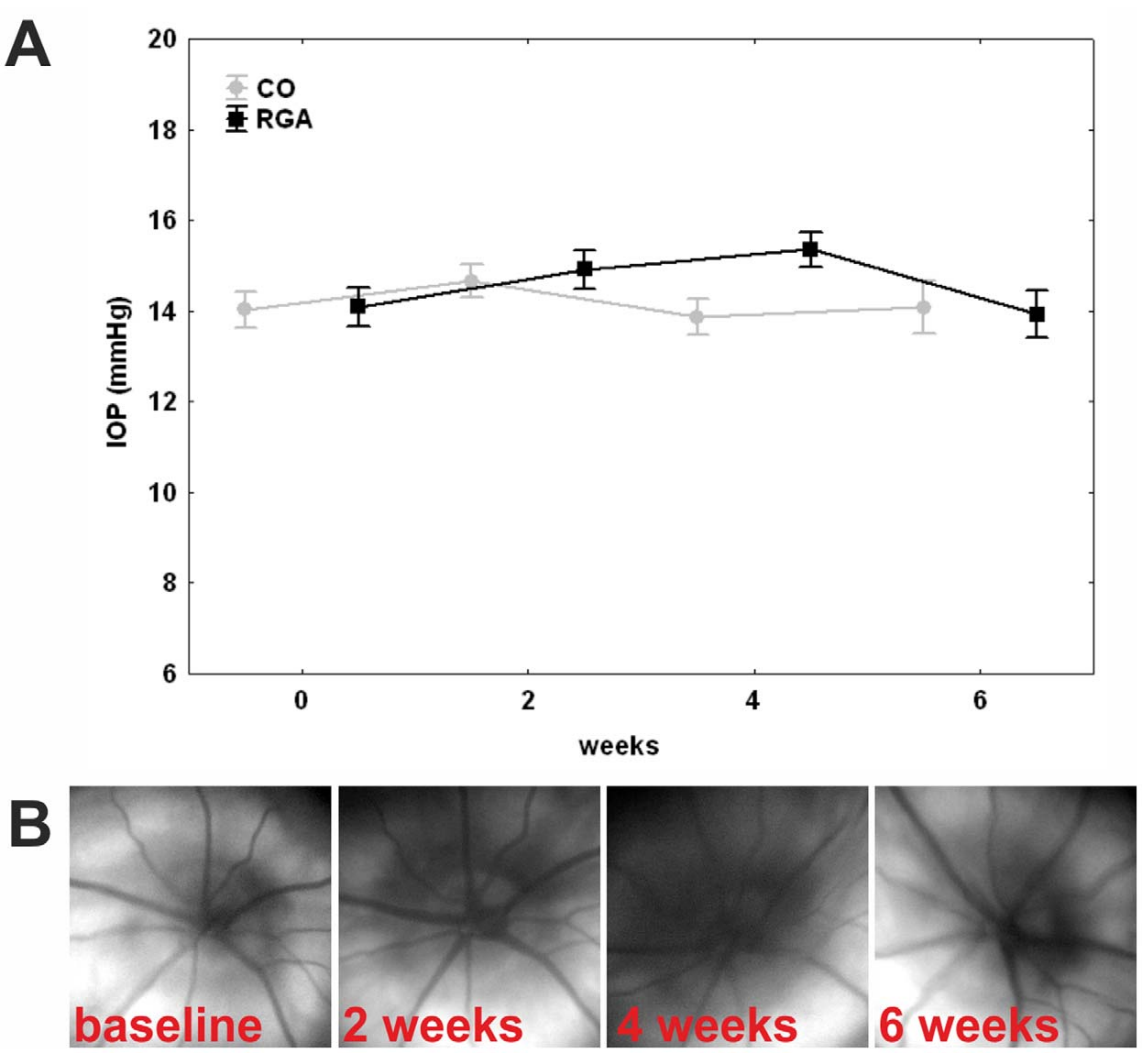
Clinical parameters. (A) Intraocular pressure in control (CO) and RGA animals. IOP was measured before (0) as well as two, four, and six weeks after immunization. There was no significant difference between groups at any point in time. (B) Fundus photos of an animal immunized with RGA at baseline as well as two, four, and six weeks after immunization. No abnormalities were observed throughout the study. Values are mean±SE.

Intraocular pressure (IOP) was consistent in the animal group immunized with the retinal ganglion cell-layer homogenate (RGA) and the control group (CO) throughout the study. A mean pressure (±SE) of 14.0±0.4 mmHg was recorded in control animals and of 14.1±0.4 mmHg in RGA animals before immunization. Mean IOP remained around 14 mmHg during the study. At six weeks the mean IOP was 14.0±0.5 mmHg in the RGA group compared to14.1±0.6 mmHg in controls (p = 0.99, fig. 1 A). No retinal detachment or bleeding was observed during fundus examinations. The optic disc appeared without pathological findings in all animals (fig. 1 B). It could be confirmed that no clinical manifestation of EAE occurred in immunized animals. The EAE score was 0 for all animals throughout this study. Immunization of rats, e.g. the Lewis strain, with certain CNS proteins is known to induce clinical signs of EAE, similar neurological deficits known from patients with multiple sclerosis [25]. These deficits usually reach their peak level between 9 and 20 days post-immunization.

### Retinal Ganglion Cell Loss

**Figure 2:**
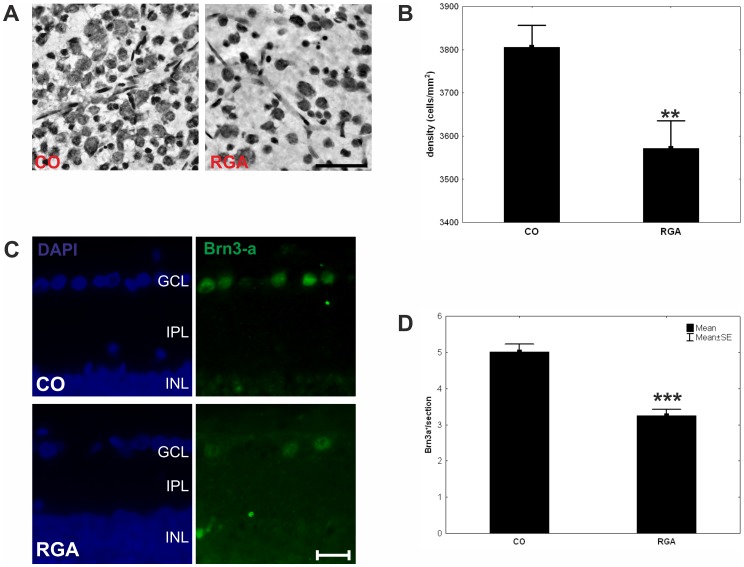
Retinal ganglion cell numbers. (A) Flatmount sections of a control (CO) and an RGA animal after cresyl stain. A lower neuron density was observed RGA flatmounts. (B) Mean retinal ganglion cell density in the control (CO) and RGA group at six weeks. (C) Exemplary Brn3-a stained retina cross-sections of a control (CO) and an RGA animal. (D). Mean number of Brn3-a^+^ retinal ganglion cells in both groups. (**, p<0.01; ***, p<0.001; scale bar in A: 10 µm and in B: 20 µm.).

In the RGA group a mean neuron density of 3571±64 cells/mm^2^ was counted on retinal flatmounts after six weeks, whereas 3805±51 cells/mm^2^ were present in the control group (fig. 2 A), constituting a significant difference between groups (p = 0.007, fig. 2 B). Looking at central and peripheral areas separately we noticed that the neuronal loss occurred mainly in the peripheral areas of the retina.

Fewer of Brn3-a^+^ RGCs were noted in retina cross-sections of RGA animals (fig. 2 C). The number of Brn3-a^+^ cells was significantly lower in the RGA group (3.2±0.2 Brn3-a^+^ cells/section) compared to controls (5.0±0.2 Brn3-a^+^ cells/section; p = 0.0001; fig. 2 D). This confirms that the RGA immunization led to a significant loss of RGCs.

### Enhancement of Autoreactive Antibodies Against Ocular Tissues

**Figure 3:**
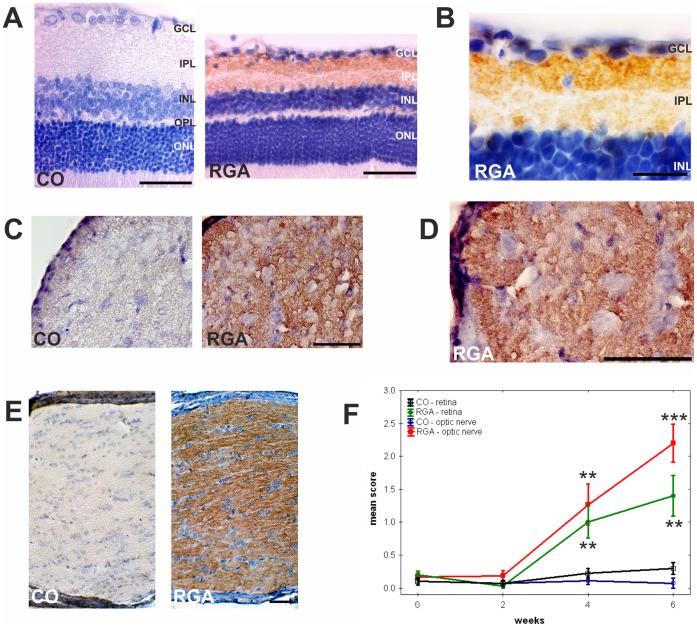
Development of autoreactive antibodies. Exemplary retina (A, B) and optic nerve cross-sections (C, D) after incubation with rat serum. Sera from RGA animals revealed a strong binding reactivity to the RGC layer and some binding to the inner plexiform layer. (E) Strong staining due to autoreactive antibodies was also observed on longitudinal optic nerve sections of immunized animals after six weeks incubated with serum from the same animal collected at the same time. (F) Development of autoreactive antibodies against ocular tissues. Time course for control (CO) and RGA animals. Values are mean±SE. Abbreviations: GCL = ganglion cell layer, IPL = inner plexiform layer, INL = inner nuclear layer, OPL = outer plexiform layer, ONL = outer nuclear layer. (**, p<0.01; ***, p<0.001; scale bars in A, C, D, E: 50 µm and in B: 20 µm.).

Animals immunized with RGA developed autoreactive IgG antibodies against ocular tissues in this study (table 1 and fig. 3). Applying a four step scoring system, ranging from 0 = no to 3 = strong staining, all animals from both groups had mean scores below 0.5 at the time of immunization (retina and optic nerve: p>0.9). At baseline the mean scores of both groups against retina and optic nerve were below 0.4 (retina: p = 0.2, optic nerve: p = 0.4). Two weeks later all animals still had low levels of autoreactive antibodies, with scores below 1 (retina: p = 0.5, optic nerve: p = 0.1). Staining for autoreactive antibodies against retinal tissue was more intense in RGA animals four weeks after immunization, with a mean score of 1.0±0.2 for the RGA group and 0.1±0.1 for the CO group (p = 0.01 fig. 3 A, B). In optic nerve sections the RGA group had a mean score of 1.3±0.3 compared to 0.1±0.1 for the CO group (p = 0.003 fig. 3 C, D). Staining intensity for autoreactive antibodies continually increased for retina sections with a mean score of 1.4±0.3 for the RGA group compared to 0.3±0.1 for the control group after six weeks (p = 0.004; fig. 3 F). A similar response was observed for optic nerve tissue with a mean score of 2.2±0.3 for the RGA group and 0.1±0.1 for the CO group (p = 0.000003; fig. 3 F). Confirmatory experiments using serum obtained at six weeks from RGA animals revealed a strong staining intensity for autoreactive antibodies against the optic nerve from the same animal (fig. 3 E).

**Table 1:** Detection of autoreactive antibodies against ocular tissues.

A. Retina			
weeks	CO	RGA	p-value
0	0.1±0.1	0.2±0.1	0.20
2	0.1±0.1	0.03±0.03	0.49
4	0.2±0.1	1.0±0.2	**0.01**
6	0.3±0.1	1.4±0.3	**0.004**
**B. Optic nerve**			
weeks	CO	RGA	p-value
0	0.1±0.1	0.2±0.1	0.39
2	0.1±0.1	0.2±0.1	0.14
4	0.1±0.1	1.3±0.3	**0.003**
6	0.1±0.1	2.2±0.3	**0.000003**

Mean scores of autoreactive antibodies during the study. Levels of antibodies against retina (A) and optic nerve (B) continuously increased in sera of RGA animals after immunization in comparison to controls (CO). At four and six weeks significantly higher antibody reactivity was detected in the RGA group. Values are mean±SE; p<0.05 in bold.

### IgG Deposition in the Retinal Ganglion Cell Layer

**Figure 4:**
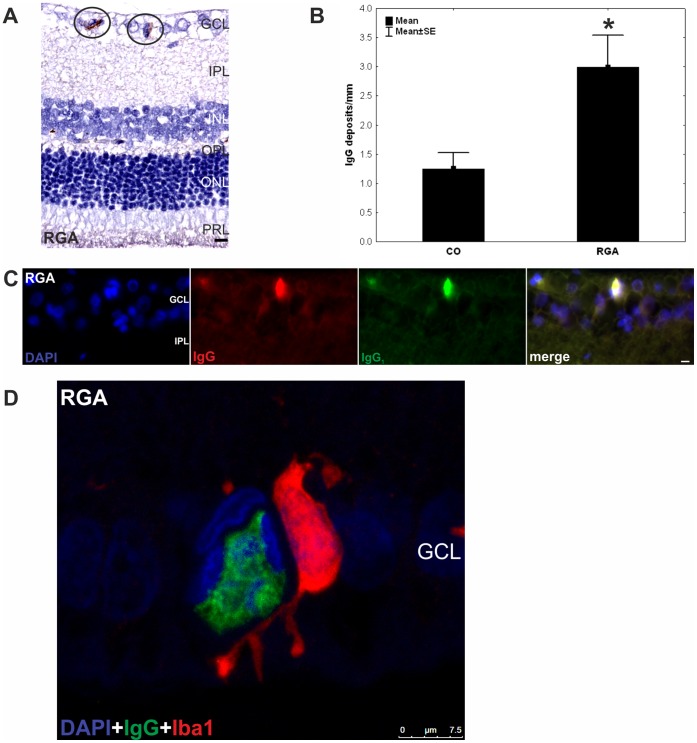
IgG deposits in the ganglion cell layer. (A) Deposits were observed in retina cross-sections (n = 10 eyes/group) of RGA animals (circled) and (B) counts revealed a significantly higher number of these deposits in the RGA group in relation to controls (p = 0.02). (C) DAPI, IgG, and IgG_1_ staining in a representative retina cross-section of a RGA immunized animal. Co-localization of IgG and IgG_1_ is evident in the merged rightmost picture. The majority of the detected deposits were of the IgG_1_ subtype. (D) Confocal image of the retina of an RGA animal. A colocalisation of the IgG deposit and an Iba1^+^ microglia can be seen. Values are mean±SE. Abbreviations: GCL = ganglion cell layer, IPL = inner plexiform layer, INL = inner nuclear layer, OPL = outer plexiform layer, ONL = outer nuclear layer, PRL = photoreceptor layer. (*, p<0.05; scale bars in A and C: 10 µm and in D: 7.5 µm).

More IgG deposits were observed in the retinas of the RGA group (3.0±0.5/mm) in relation to the control group (CO: 1.3±0.3/mm, p = 0.02, fig. 4 A and B). An increased amount of IgG deposits, predominantly of the IgG subtype 1, was noted especially in the retinal ganglion cell layer of RGA immunized animals (fig. 4 C). We did not note any other discernable changes in any retinal layer.

### Colocalisation of IgG Deposition and Microglia

**Figure 5:**
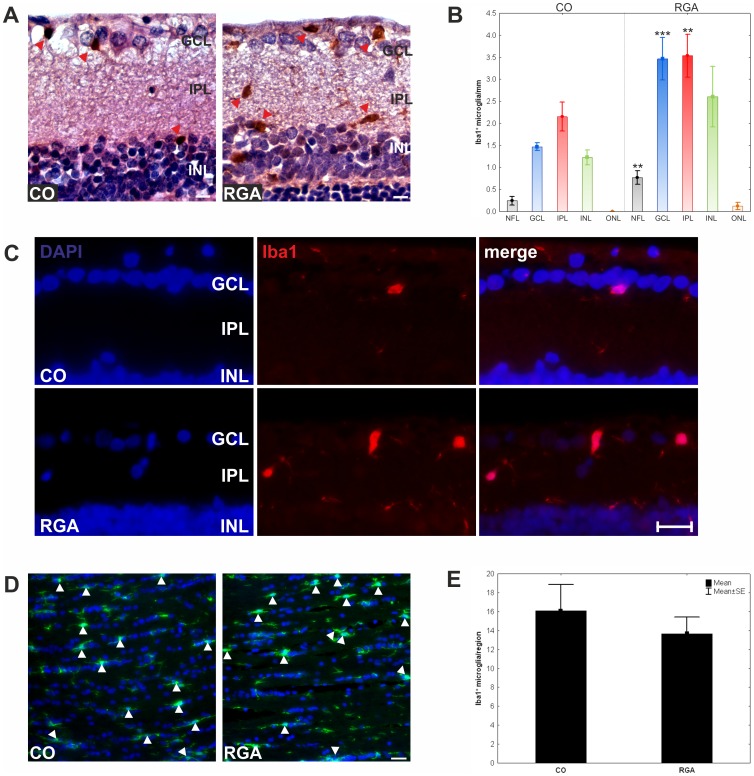
Examination of microglial cells in the retina and optic nerve. (A) Exemplary Iba 1^+^ stained microglia in retina cross-sections of the RGA and the CO group were observed (red arrowhead). (B) Mean number of Iba 1^+^ microglia in the different retinal layers. Significantly higher numbers of Iba 1^+^ microglia were noted in the NFL, GCL, and IPL. (C) Exemplary immunofluorescence stained retina cross-sections. For both groups (RGA, CO) the DAPI, the Iba 1^+^ microglia, and the merge picture are shown. (D) Optic nerves of control animals had fewer Iba 1^+^ microglia cells (arrowheads) then RGA animals, but more resting microglia (arrows). (E) There was no significant difference in the total number of Iba1^+^ microglia in optic nerves of the RGA and the CO group. Values are mean±SE. Abbreviations: GCL = ganglion cell layer, IPL = inner plexiform layer, INL = inner nuclear layer, OPL = outer plexiform layer, ONL = outer nuclear layer. (**, p<0.01; ***, p<0.001; scale bars in A: 10 µm and in C and D: 20 µm).

A significantly higher number of IgG deposits and Iba^+^ microglia was noted in retinas of the RGA group (fig. 4 and 5). These two findings were often colocalised. In fig. 4 D a microglia cell is shown that literally surrounds the IgG deposition with its cytoplasmic extensions. The nucleus within the deposit is already split into several fragments. This could indicate that this microglia was more cytotoxic active.

### Analysis of Microglia Cells

The number of Iba1^+^ microglial cells in the retina and optic nerve was evaluated six weeks after immunization.

#### Retina

A significantly higher number of Iba1^+^ microglia was observed in RGA retinas (RGA: 10.5±1.5 cells/mm, CO: 5.1±0.4 cells/mm; p = 0.003; fig. 5 A). The highest number of microglia were observed in the retinal ganglion cell layer (RGA: 3.5±0.5 cells/mm, CO: 1.4±0.1 cells/mm; p = 0.001) and the inner plexiform layer (RGA: 3.5±0.5 cells/mm, CO: 2.2±0.3 cells/mm; p = 0.03; table 2 and fig. 5 B and C).

#### Optic nerve

Regarding the optic nerve sections, no significant difference in the total number of Iba1^+^ microglia was observed between the RGA (13.7±1.7) and the CO (16.1±2.8) group (p = 0.5; fig. 5 C-D).

### Retinal Protein Levels of Microglial and Macrophage Markers

Antibody microarray analysis using microglia and macrophage markers revealed higher protein levels in retinas of RGA immunized animals compared to controls. The level of Iba1 (+18%; RGA: 7065±1175U_art_, CO: 5960±1506U_art_; p = 0.03) and MHC class I (+13%; RGA: 12934±1048U_art_, CO: 11425±1034U_art_; p = 0.01) was significantly increased in retinas of RGA animals. A slight increase was also noted regarding CD68 (+11%; RGA: 14966±11855U_art_, CO: 13463±1782U_art,_ p = 0.17), CD11b (+13%; RGA: 11668±1957U_art_, CO: 10307±1831U_art_, p = 0.27), and MHC class II 1a (+12%; RGA: 8269±936U_art_, CO: 7364±935U_art_, p = 0.06) levels in the RGA group. Moreover, these results confirmed the immunohistochemistry of the enhanced amount of Iba^+^ microglia in the retina.

### GFAP Immunohistochemistry

**Figure 6:**
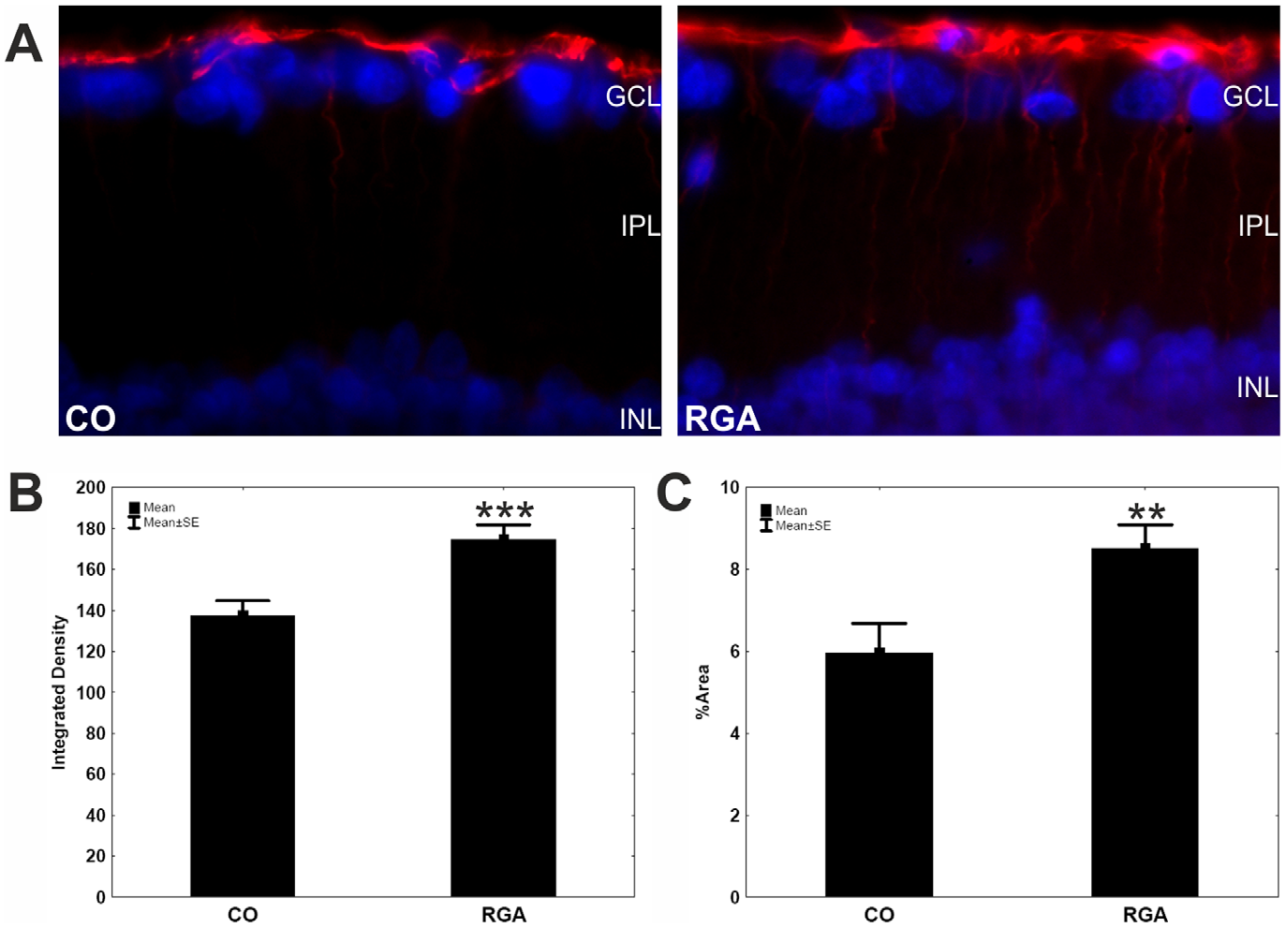
GFAP stain in retina sections. (A) GFAP expression (red) in representative retina cross-sections of a control (left) and a RGA animal (right) at six weeks. A significantly higher GFAP staining intensity given as integrated density (B) as well as an enhanced %Area of GFAP^+^ staining (C) was detected for the RGA group in comparison to controls Values are mean±SE. Abbreviations: GCL = ganglion cell layer, IPL = inner plexiform layer, INL = inner nuclear layer. (**, p<0.01; ***, p<0.001; scale bar: 10 µm).

To investigate whether gliosis occurs in our model of autoimmune glaucoma, we stained retina cross-sections with anti-GFAP antibody. In control retinas, GFAP immunoreactivity was restricted mainly to the ganglion cell layer. An increased GFAP labeling was observed six weeks after immunization (fig. 6 A). These cells were stained along their entire length. The integrated density (fig. 6 B) and the GFAP^+^ area ( = %Area; fig. 6 C) were significantly increased in the RGA group (integrated density: 174.7±41.9; %Area: 8.5±3.4) compared to controls (integrated density: 137.6±36.8, p = 0.0006; %Area: 5.9±3.6, p = 0.006).

### GLAST Immunohistochemistry

**Figure 7:**
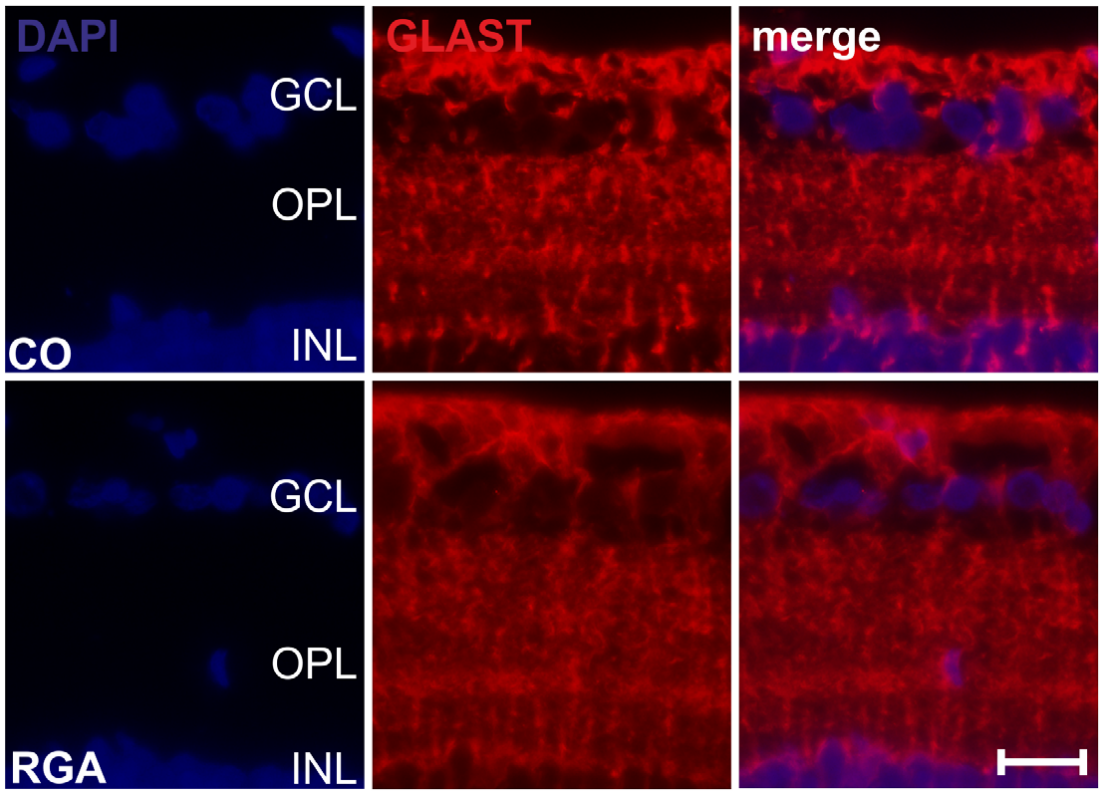
GLAST stain in retina sections. Exemplary retina cross-sections of an control (CO) and an RGA animal stained with GLAST six weeks after immunization. A similar EAAT1 distribution was noted in both groups. (scale bar: 20 µm.).

GLAST immunoreactivity appeared to be comparable in RGA and control retinas (fig. 7), no differences between the groups were noted. GLAST stain was diffuse in all retinas, including GCL, OPL and INL.

### Optic Nerve Demyelination

**Figure 8:**
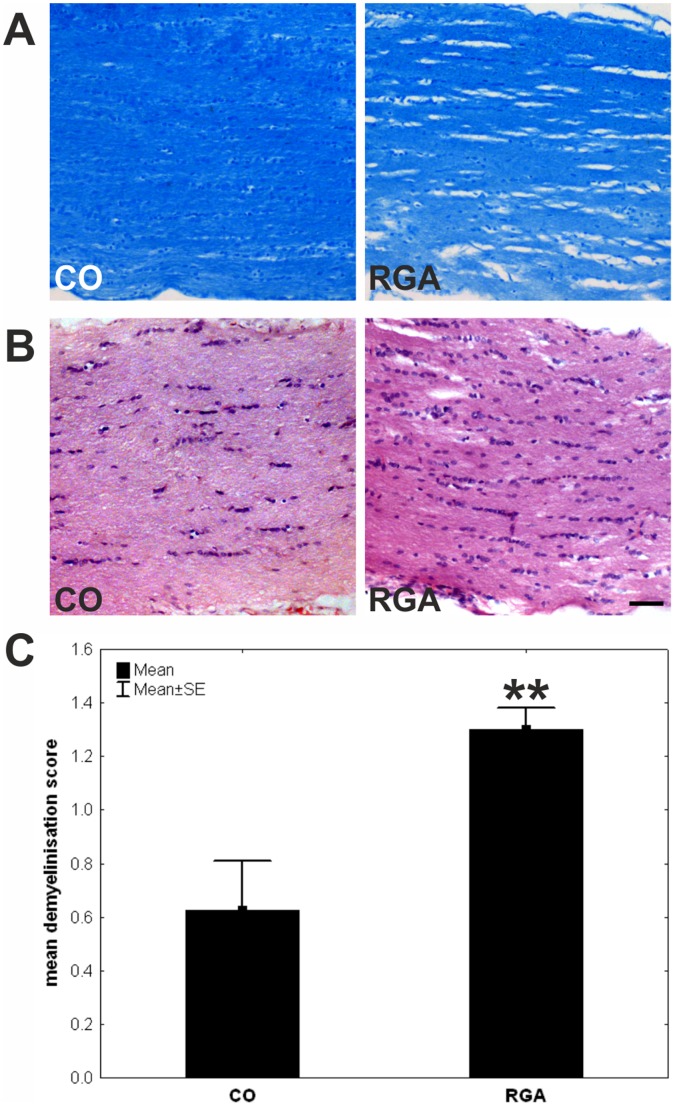
Examination of optic nerves. Exemplary LBF/Nissl (A) and H&E (B) stained longitudinal optic nerve sections of a control (CO) and an RGA animal six weeks after immunization. (C) Immunized animals showed significantly more signs of demyelination. Values are mean±SE. (**, p<0.01; scale bar: 50 µm).

After LFB and LFB/Nissl staining, alteration and disruption in the organization of the myelin sheath was observed in the immunized group. The optic nerves of control animals showed no or little signs of demyelination and had intact lamellar structures (fig. 8 A). The mean demyelination score was 1.30±0.48 for the RGA group and 0.62±0.51 for the control group (fig. 8 C), with a significant group difference (p = 0.002). This indicates a significant demyelination after RGA immunization. Stronger infiltrates or additional pathological effects were not noted on optic nerve sections after H&E staining (fig. 8 B).

**Table 2:** Distribution of iba1^+^ microglia in retina.

retinal layer	CO	RGA	p-value
nerve fiber layer	0.2±0.1	0.7±0.2	**0.01**
retinal ganglion cell layer	1.4±0.1	3.5±0.5	**0.001**
inner plexiform layer	2.2±0.3	3.5±0.5	**0.03**
inner nuclear layer	1.2±0.2	2.6±0.7	0.06
outer nuclear layer	0	0.1±0.1	0.14

Number of Iba1^+^ somas counted in each retinal layer. Significantly higher numbers Iba1^+^ microglia were present in the nerve fiber, ganglion cell and inner plexiform of retinas from RGA immunized animals. Values are mean±SE; p<0.05 in bold.

### Brain Sections

Brain sections were investigated after LFB/PAS and H&E staining. Observations of the CNS revealed no pathological aberrations, such as inflammatory infiltrates, demyelination or hemorrhages, which are known for example from brains of animals with experimental autoimmune encephalitis (EAE). Sections of the brain stem, cerebellum and hippocampus from all animals were examined and showed no lesions (fig. S1). The anterior part of the optic tract was intact in all sections where it could be evaluated.

## Discussion

In this study we observed a significant reduction of RGCs after immunizing rats with an RGC layer homogenate. The development of antibodies against ocular tissue in serum from these rats (fig. 3) and the occurrence of IgG deposits in their retinas (fig. 4) suggest a substantial involvement of antibodies in the processes leading to this cell death. Moreover, a higher level of Iba1^+^ microglia could be detected in RGA retinas (fig. 5 A–C). Moreover, significantly increased levels of Iba1 were also detected in RGA retinas using protein microarrays. These findings could indicate a cellular alteration, which probably favored cell death.

Antibodies are considered relevant pathogenic factors in neurodegenerative diseases [26], [27] including glaucoma [28]. In multiple sclerosis, an inflammatory demyelinating disorder of the central nervous system, it has been proposed that autoantibodies to neuronal proteins could mediate tissue damage. Antibodies binding to cell-surface receptors might act as agonists or antagonists [29], potentially contributing to axonal damage and neuronal loss [30]. Myelin specific antibodies, in the presence of complement, can induce rapid demyelination [31]. Possibly axon-specific antibodies induce axonal damage. In an animal model of a neurodegenerative disorder of the neuronal ceroid lipofuscinosis group, the Battens disease, IgG depositions occur in the CNS [32]. Additionally, a compromised blood-brain-barrier was observed, which supports the possibility of systemic produced antibodies being able to enter the CNS. But also in studies regarding eye tissues, autoantibodies were held responsible for the RGCs death, observed after immunization with heat shock proteins [23], [24]. It has been proposed that HSP 27 antibodies could enter neuronal cells via endocytosis and lead to subsequent apoptotic cell death [28]. We hypothesize that similarly to this mechanism autoreactive antibodies produced after immunization in this model, pass the damaged blood-retina barrier and are able to access ocular structures. Then, through their binding to their superficial or intracellular (after a possible endocytosis) antigenic targets, they lead to RGC apoptosis. The detection of IgG deposits in the RGC layer after immunization is a further indicator for this mechanism (fig. 4). Detailed examination of the possible binding sites of antibodies in our model could further confirm similar observations.

We propose that these depositions are due to a generalized immune dysregulation and are not produced locally. Detailed examination of the possible binding sites of antibodies in our model could further confirm similar observations. In addition, autoantibodies are able to mediate a so called antibody dependent cellular cytotoxicity via microglia in the CNS [33], [34]. This microglial cytotoxicity is characterized by local immune modulation via chemokine, but mainly through secretion of TNF-alpha and Fas-ligand [35], [36]. There are is some evidence that pathway leading to RGC death in this model could by via indirect activation of TNF-alpha or Fas-ligand [24]. Furthermore, we noted microglia, co-localized with IgG antibody depositions in the retinal ganglion cell layer (fig. 4 D), which is an hint, that microglia have a important contribution in the pathogenesis of this model [37].

The development of autoantibodies, confirmed through the presence of antibody deposits on retinal tissues and autoreactive antibodies, should be regarded as an aspect of a generalized immune dysregulation, caused by the immunization, and not as a local event. In this aspect it has to be noted that this generalized immunization did not induce a generalized neurodegeneration. No pathologic findings were observed in CNS tissues and animals were without detectable neurological deficits throughout the study. These findings speak in favor of a primarily ocular specific damage in this model.

Antibodies in this model of autoimmune glaucoma could produce profound abnormalities without interacting with other elements of the immune system and could disrupt cellular function by binding to intracellular or extracellular sites [19].

In our study, animals were immunized with a retinal ganglion cell layer homogenate, which besides RGCs contains amacrines [38] and non-neuronal cells like astrocytes. Therefore, the observed effects might not be based on RGCs alone.

Since complex alterations in autoantibody patterns are detectable in glaucoma patients [7], [39], it is likely that not just one specific antibody is involved in triggering RGC death. Therefore, we used a homogenate of RGC layer, a rather complex mixture of proteins, as the immunizing antigen.

Apart the possible role of antibodies in RGCs death, the behavior of glia cells in our study was also examined. It is known that human glaucoma as well as animal models of elevated intraocular pressure alter GFAP immunoreactivity in the retina [15]. We observed gliosis due to GFAP hypertrophy in RGA immunized animals (fig. 6). While a similar GLAST expression was noted in both groups. This finding is in accordance with other reports on stable GLAST expression in retinas of glaucomatous eyes [40].

Microglia activation has been reported to occur in retina of glaucoma animal models including DBA/2J or models of elevated intraocular pressure [41]. After partial optic nerve transsection a dynamic migration of microglia toward a lesion site could be noted [42]. Microglial activation is today considered to be more complex than an all-or-nothing process [43]. Depending on the stimulus and biochemical milieu, a wide range of qualitatively varied responses, such as pro- and anti-inflammatory, neuroprotective or neurotoxic, may be elicited [44]. In RGA immunized animals the highest levels of microglia were noted in the NFL, GCL, and IPL (table 2 and fig. 5 A).

In summary, the RGC death in this model is accompanied by the development of autoantibodies and IgG depositions. Alterations in microglia levels and GFAP hypertrophy are likely a consequence of the observed cell death.

## Materials and Methods

### Animals

All procedures concerning animals adhered to the ARVO statement for the use of animals in ophthalmic and vision research. All experiments involving animals were approved by the animal care department of Rhineland-Palatinate, Germany.

Male Lewis rats (Charles River), six weeks of age, were used for the experiments and kept under environmentally controlled conditions with free access to chow and water. Detailed observations and health checks including eye exams and scoring for clinical signs of experimental autoimmune encephalitis were performed regularly.

### Antigen Preparation and Immunization

A homogenate of bovine retinal ganglion cell layer was used for immunizations. Therefore, a pull-off approach to separate the ganglion cell layer from the rest of the retina was used [45], [46]. The anterior part of fresh bovine eyes, obtained from the local abattoir (Schlachthof Alzey, Alzey, Germany), was dissected along the ora serrata followed by vitreous removal. Retinas were placed on filter paper (Millipore), with the retinal ganglion cell-layer on top, incubated and then rinsed with PBS. Superfrost Plus slides (Thermo) were placed on the nerve fiber layer. Slides were removed within the adherent nerve fiber and ganglion cell layer. The preparations were always made under microscopic observation [46]. To confirm a successful separation some samples were shortly stained with hematoxylin and cresylblue and examined microscopically. Ganglion cell layers were were homogenized after freezing in liquid nitrogen. The pulverized RGC layer tissue was re-suspended in PBS and stored at -80°C until used for injections.

Animals were immunized with 8 mg retinal ganglion cell layer homogenate in an equal volume of incomplete Freund’s adjuvant plus 3 µg pertussis toxin (both Sigma). They were always compared to an equal group of control animals (CO), these animals were injected with NaCl in Freund’s adjuvant and pertussis toxin.

### Intraocular Pressure Measurement and Fundus Photography

IOP was measured using a TonoPen® XL applanation tonometer (Medtronic). Ten measurements per eye were done before immunization and every two weeks after. Fundus pictures were taken through a surgical microscope (Zeiss) at the same time.

### Evaluation of Ganglion Cell Numbers

#### Retinal flatmounts

Eyes were enucleated and fixed in 4% paraformaldehyde (Sigma). One eye per animal was used for retinal flatmounts (n = 10 eyes/group). After de- and rehydration in 70% to 100% ethanol, retinal flatmounts were stained using the Nissl staining with 2% cresylblue (Merck) [47], [48]. Subsequently all slides were again dehydrated in ethanol followed by incubation in xylene (Merck), before flatmounts were mounted with Eukitt (Merck). Eight pictures were taken using 100x magnification for cell counts. Four predefined areas were arranged circularly around the optic nerve head. The outer four areas were located edging the beginning of the ora serrate.

All pictures were taken with a CCD camera attached to a microscope (Vanox-T). All cells in the RGC layer were manually counted by a masked examiner using a cell counter plug-in (ImageJ; NIH). The Nissl staining allowed the distinction between neurons and glia based on their morphology, location, and size [38], [49], [50]. Endothelial cells were not included in the further analysis. Cells with rich Nissl substance in irregular outlines, a prominent nucleolus, and a minimum diameter size of 8 µm were classified as neurons. Glial cells were classified as follows: diameter <8 µm, commonly round with regular outlines, and much darker staining.

#### Cross-sections

Eyes were embedded in paraffin and retinal cross-sections were cut and mounted on Superfrost Plus slides (Thermo). Cross-sections (5 µm) from control and RGA animals (6 eyes/group; 6 sections/eye) were deparaffinized and antigen retrieval was performed by heating slices for 20 min in citrate buffer (95°C). The sections were blocked with 10% normal donkey serum in 0.1% Triton-X in phosphate buffered saline for 1 h. Sections were then incubated overnight at room temperature with a goat polyclonal Brn3-a antibody (1∶100; Santa Cruz; Germany) [51] followed by donkey anti-goat Alexa Fluor 488 (1∶400; Dianova, Germany) secondary antibody. All slides were mounted with antifade medium with fluorescent nuclear stain DAPI (Fluoro-Mount w/DAPI; Dianova; Germany). Microscope images of the periphery, middle and central part of the retina of each section were digitally captured with an Axiocam HRc CCD camera on a Zeiss Imager M1 fluorescence microscope using a 40x objective. The digitalized images were transferred to Corel PaintShop Photo Pro (Ver. 13; Corel Corporation; CA; USA). Excerpts of the GCL were taken from the pictures and RGCs were counted in the GCL using ImageJ software.

### Detection of Autoantibodies Against Ocular Tissue

Autoreactive antibodies against retina and optic nerve were detected at different points in time. An adapted protocol previously used to detect circulating autoantibodies against brain [52], [53]_ENREF_34 and retinal tissue [54], [55] was applied. Blood was drawn via tail vein puncture prior as well as two and four weeks after immunization (n = 10 samples/point in time). A final blood sample (at six weeks) was collected via heart puncture [56].

Following enucleation, eyes and optic nerves from healthy Lewis rats were fixed in 4% formaldehyde and embedded in paraffin. 1 µm thick retina and optic nerve cross-sections were cut. Sections were treated with xylol, rehydrated with decreasing alcohol concentrations and pretreated with 0.3% hydrogen peroxide. Subsequently incubation with target retrieval solution (Dako) and 1% bovine serum albumin followed. Sections were incubated with rat sera (retina: 1∶200; optic nerve: 1∶750; in PBS). This step was followed by incubation with amonoclonal anti-rat IgG secondary antibody (H+L; 1∶500; Pierce). Bound antibodies were detected with diaminobenzidine staining (DAB; DCS, Hamburg, Germany) and sections counterstained with hematoxylin. All tissue sections were scored by three independent masked examiners through a microscope, using the following scoring system: 0 = no DAB staining; 1 = weak staining; 2 = moderate staining and 3 = strong staining [52], [56], [57].

### Immunohistochemical Labeling of Retina Cross-sections

Retina cross-sections were cut (3 µm) and treated as described above.

In order to visualize possible retinal antibody deposits (six weeks after immunization) anti-rat IgG antibody (1∶500; Immunopure) and a subsequent DAB staining was applied to retina cross-sections (n = 10 eyes/group). Hematoxylin counterstain was used and deposits were counted by three examiners in a masked fashion on all sections.

In order to differentiate between IgG subtypes additionally retina sections were co-labeled with goat anti-rat IgG TRITC (1∶100) and goat anti-rat IgG_1_, IgG_2a_, IgG_2b_, and IgG_3_ FITC (1∶400, all GenWay) antibodies overnight. Tissue was covered with Vectashield containing diamidin-2-phenylindol (Vector) the next day. Images were acquired through a fluorescent microscope (TE 2000) as described above.

The ionized calcium binding adaptor molecule 1 (Iba 1) was used as microglial marker [58], [59]. Microglia were visualized on sagittal cross-sections of the eyes (3 µm; n = 7 eyes/group) and longitudinal sections of the optic nerve (n = 5 nerves/group) with anti-Iba 1 primary antibody (1∶500; Wako Pure Chemical Industries; Neuss, Germany). Regarding retina cross-sections, this step was followed by goat-anti rabbit HRP conjugated secondary antibody (1∶400, Calbiochem, Darmstadt, Germany) treatment, DAB incubation, and hematoxylin counterstain. For quantification, Iba^+^ somas were counted in the retinal layers. Numbers of microglia were put in relation to retinal length (cells/mm) and the cell location was also recorded. For immunofluorescence microscopy of the retina and optic nerve sections, a Cy3 conjugated IgG secondary antibody (1∶500; Linaris) was applied. For cell counts on optic nerve sections images were obtained via a TE 2000 microscope as described above. The camera field was placed on the optic nerve at the transition to the chiasma opticum. Only cells with positive Iba1 staining and a DAPI marked nuclei in the center were counted in the region of interest (region: 244×244 µm).

GFAP reactivity on cross-sections (n = 7 eyes/group; cut through the optic nerve head) was visualized via rabbit anti-GFAP antibody (1∶1000, Sigma-Aldrich) followed by a Cy3 conjugated goat anti-rabbit IgG secondary antibody (1∶500, Linaris). Four images of each retina (2 central and 2 peripheral ones) were acquired through a fluorescent microscope (TE 2000; Nikon) equipped with a CCD camera (60x magnification). All slides were previewed and optimum exposure time and gain was calculated for a standardized data acquisition. A computer assisted quantification method was applied for GFAP analysis. GFAP intensity ( = integrated density) [15], [60] and a comparison for the percentage of GFAP^+^ stained area ( = %Area) [60], [61] were evaluated on all slides. A software based processing with standardized background subtraction (rolling ball algorithm), threshold setting and analysis was applied to the digital images using macros in ImageJ.

Glutamate/aspartate transporter (GLAST) was labeled on retina cross-sections (n = 6 eyes/group) using rabbit polyclonal GLAST antibody (1∶250; Abcam; UK). Donkey anti-rabbit Alexa Fluor 555 (1∶500; Invitrogen; NY, USA) was applied as secondary antibody. The sections were mounted with antifade medium with fluorescent nuclear stain DAPI (Fluoro-Mount w/DAPI; Dianova; Germany).

Additionally, several retina cross-sections from both groups were cut in 15 µm thicknesses and incubated with anti-rat IgG and anti-Iba 1 antibody overnight. The next day sections were incubated with fluorescent secondary antibodies and nuclei were counterstained with DAPI. Sections were imaged using a confocal microscope (TSC SP5; Leica Microsystems; Mannheim; Germany).

### Histopathology of the Optic Nerve

Optic nerves were dissected at the chiasma opticum and 3 µm thick longitudinal sections were stained with Harris Hematoxylin and Eosin G (H&E both Merck) for evaluation of pathological aberrations (n = 5 nerves/group) and Luxol Fast Blue (LFB, Solvent Blue 38, Merck) with or without Nissl (LFB/Nissl) in order to detect possible demyelination (n = 5 nerves/group). Per animal, two slides of each stain were examined and by two observers masked to the protocol. The grade of demyelination was quantified using a 0 to 3 scoring system with 0 = no demyelination; 1 = rare foci of demyelination; 2 = a few areas of demyelination; and 3 = large areas of demyelination [62].

### Brain Sections

Horizontal serial brain sections (3 µm), obtained twelve days (n = 5 per group) and six weeks (n = 10 per group) after immunization, were stained for myelin sheaths with LFB using Kluver’s modified method. The counterstaining was performed with the periodic acid Schiff staining. Additional brain sections (n = 10 per group) were investigated for pathological aberrations after standard H&E staining.

### Antibody Microarrays

In order to confirm the immunohistochemical observations regarding microglial activation, an antibody microarray approach [63] was performed using retinal lysates from both groups (n = 7/group). To quantify specific protein levels, which are associated with microglial activation and macrophagic properties, the setup contained antibodies against Iba1, CD11b (OX42), CD68 (ED1), MHC class I (OX18) and MHC class II 1a (OX6; table S1). These antibodies were spotted as triplicates on nitrocellulose slides (Oncyte, Grace Bio-Labs; Bend; USA) using a non-contact array spotter (sciFLEXARRAYER 3; Scienion; Berlin; Germany).

Retinal tissues, frozen in liquid nitrogen, were homogenized and resuspended in 100 µl 0.1% SDS/PBS. After the measurement of the total protein concentration using a bicinchoninic acid protein assay (BCA kit; Thermo), 7.5 µg of protein were labeled with 0.45 µl of a fluorescent dye (DyLight®649; Thermo). Microarray slides were subsequently incubated with the labeled protein extracts for 2.5 h at 4°C on a rocking platform. Afterwards, slides were scanned with a high-resolution confocal scanner. Spot intensities were quantified on 10 dB images using Spotfinder Tiger software (Vers. 3.1.1; Software TM4; Dana-Faber Cancer Institute; Boston; MA; USA). For further analysis, the local background signal was subtracted from the median signal of each spot. The raw data were globally normalized using a factorization to constant scale intensity (20000 U) for each subarray. The individual normalization coefficients were applied and raw intensities were converted artificial intensities (U_art_) for each antibody reactivity. Subsequently, intensity of the three technical replicates for each antibody were averaged and used for the statistical analysis.

### Statistics

Data are presented as mean±SE. Regarding histology data the two groups were compared using two-tailed Student t-test (Statistica V8.0; Statsoft). The antibody array data was compared using nonparametric Mann-Whitey U test, U_art_ values are given as mean±SE. Null hypotheses were rejected at p<0.05.

## Supporting Information

Figure S1 **Brain histology.** (A) Representative brain sections of control (CO) and RGA animals stained with LFB/PAS 12 days after immunization. (B) Six weeks after immunization brain sections of control (CO) and RGA animals were stained with H&E and LFB/PAS. At both points in time all brain sections were without pathological findings. (scale bar: 100 µm.)
(TIF)

Table S1 **Antibodies used for microarray detection.**
(DOCX)
