# Supplementary figures and images for: Retinal Ganglion Cell Loss Is Accompanied by Antibody Depositions and Increased Levels of Microglia after Immunization with Retinal Antigens
Source: PLoS One. 2012 Jul 26;7(7):e40616. doi: 10.1371/journal.pone.0040616 (PMC3406064; doi:10.1371/journal.pone.0040616)

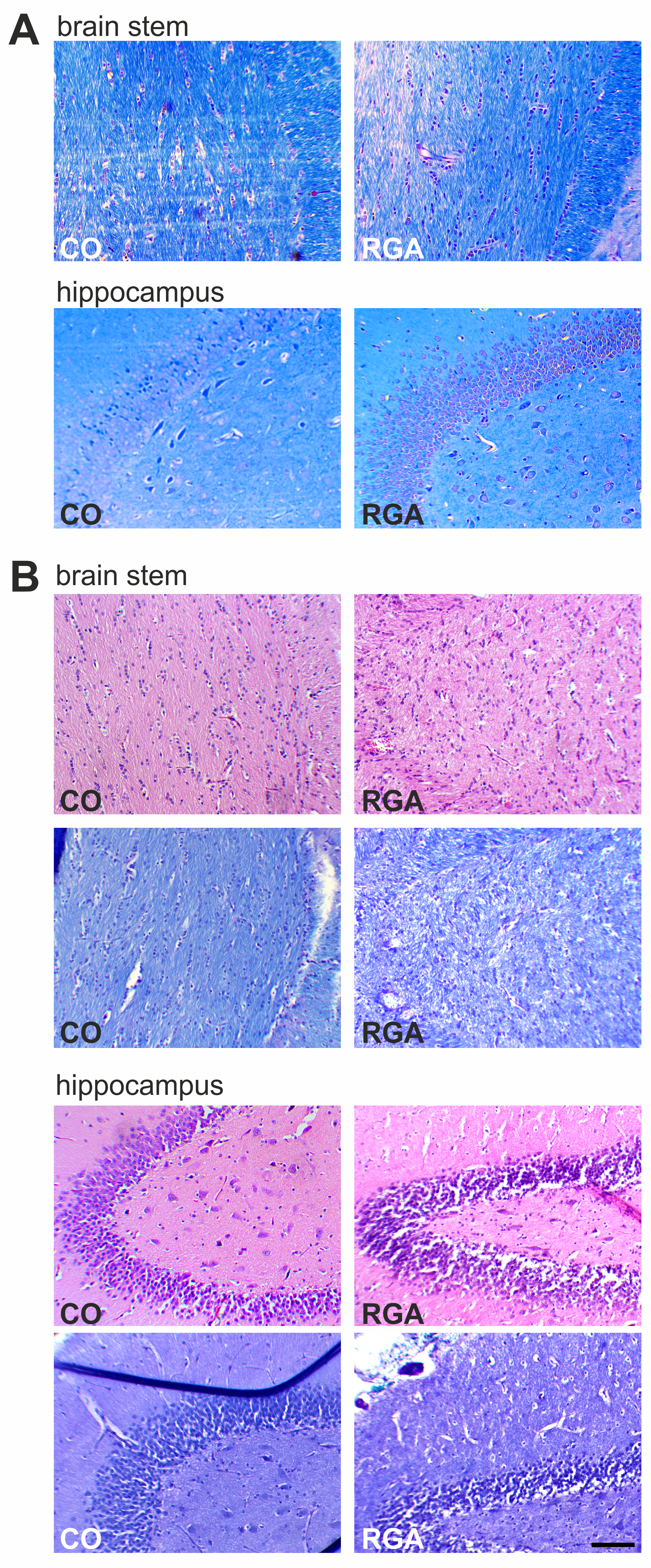

Supplement: Figure S1 — Brain histology. (A) Representative brain sections of control (CO) and RGA animals stained with LFB/PAS 12 days after immunization. (B) Six weeks after immunization brain sections of control (CO) and RGA animals were stained with H&E and LFB/PAS. At both points in time all brain sections were without pathological findings. (scale bar: 100 µm.) (TIF) [file pone.0040616.s001.tif]
