# Supplementary material for: Retinal Ganglion Cell Loss Is Accompanied by Antibody Depositions and Increased Levels of Microglia after Immunization with Retinal Antigens
Source: PLoS One. 2012 Jul 26;7(7):e40616. doi: 10.1371/journal.pone.0040616 (PMC3406064; doi:10.1371/journal.pone.0040616)
Supplement: Table S1 — Antibodies used for microarray detection. (DOCX) [file pone.0040616.s002.docx]

**Table S1. Antibodies used for microarray detection.**

| antibody | detection of | concentration | supplier |
| --- | --- | --- | --- |
| Iba1 | microglia, macrophages | 1 mg/ml | Wako Pure Chemicals, Neuss, Germany |
| CD11b (OX42) | microglia, macrophages | 1 mg/ml | Abcam, Cambride, UK |
| CD68 (ED1) | microglia, macrophages | 1 mg/ml | Abcam, Cambride, UK |
| OX18 | MHC class I | 1 mg/ml | Abcam, Cambride, UK |
| OX6 | MHC class II 1a | 1 mg/ml | Abcam, Cambride, UK |
|  |  |  |  |

Celltypes detected by antibodies usued for micorarrays as well as their spotting concentration and their supplier.
